# Automatic Groove Measurement and Evaluation with High Resolution Laser Profiling Data

**DOI:** 10.3390/s18082713

**Published:** 2018-08-17

**Authors:** Lin Li, Wenting Luo, Kelvin C. P. Wang, Guangdong Liu, Chao Zhang

**Affiliations:** 1 College of Transportation and Civil Engineering, Fujian Agriculture and Forestry University, Fuzhou 350002, China; 2 School of Civil and Environmental Engineering, Oklahoma State University, Stillwater, OK 74078, USA; kelvin.wang@okstate.edu; 3 Fujian Provincial Expressway Technology Consulting Co., Ltd. Fuzhou 350002, China; lgd-1234@163.com (G.L.); zhangchao120061@126.com (C.Z.)

**Keywords:** airport runway, K-means clustering, groove dimension, Naïve Bayes Classifier, Support Vector Machine, point laser, profiling data

## Abstract

Grooving is widely used to improve airport runway pavement skid resistance during wet weather. However, runway grooves deteriorate over time due to the combined effects of traffic loading, climate, and weather, which brings about a potential safety risk at the time of the aircraft takeoff and landing. Accordingly, periodic measurement and evaluation of groove performance are critical for runways to maintain adequate skid resistance. Nevertheless, such evaluation is difficult to implement due to the lack of sufficient technologies to identify shallow or worn grooves and slab joints. This paper proposes a new strategy to automatically identify airport runway grooves and slab joints using high resolution laser profiling data. First, K-means clustering based filter and moving window traversal algorithm are developed to locate the deepest point of the potential dips (including noises, true grooves, and slab joints). Subsequently the improved moving average filter and traversal algorithms are used to determine the left and right endpoint positions of each identified dip. Finally, the modified heuristic method is used to separate out slab joints from the identified dips, and then the polynomial support vector machine is introduced to distinguish out noises from the candidate grooves (including noises and true grooves), so that PCC slab-based runway safety evaluation can be performed. The performance of the proposed strategy is compared with that of the other two methods, and findings indicate that the new method is more powerful in runway groove and joint identification, with the F-measure score of 0.98. This study would be beneficial in airport runway groove safety evaluation and the subsequent maintenance and rehabilitation of airport runway.

## 1. Introduction

Since the early 1960s, grooving has been increasingly used on airfield pavements and roadways to improving tire-pavement friction through efficient water-run-off and prevent airplanes or vehicles from accidents [1,2]. The purpose of airport runway grooves is to improve pavement skid resistance and reduce the occurrence of potential hydroplaning. Numerous field studies and research efforts have demonstrated the effectiveness of grooves in reducing skid resistance-related accidents on both roadways and runways [3,4,5,6,7]. Field tests indicate groove configuration dimensions are highly associated with the airplane landing and take-off safety [8,9]. In addition, hydroplaning speed prediction models have been developed to investigate the relationships between skid resistance and groove dimensions (depth, width, and spacing) [9,10]. Results indicate larger groove depth and width, and tighter groove spacing would result in better frictional properties. Particularly, in Ong’s studies [9,10] changes in groove depth are found to have the most significant effects on hydroplaning. Therefore, the periodic measurement and assessment of groove dimensions are critical for resisting hydroplaning and assuring airplane landing and take-off safety.

Past studies indicate that groove dimensions can be automatically measured using point laser ranger-based profiling equipment and 3D laser imaging sensor-based instruments [11,12,13,14,15]. For the collected grooving data, the identification can be implemented in three phases, namely the potential dip identification, the determination of two endpoints of the identified dip, and true groove determination.

Currently there are four methods for the potential dip identification [11,12,14,16]. One method is the cluster-based technique, whose identification results are inconsistent at the different field test sites. This method is unsuitable for the measurement of abrasive or worn grooves since the groove identification depends on the cluster analysis results of profile slopes [14]. In addition, this method requires setting a threshold to distinguish outside-groove points and inside-groove points. Another method is the low-pass based technique [12,16], a smoothed profile is produced by applying the low-pass filter in the raw profile data, and the difference between the smoothed profile and raw profile is used to identify the grooves. This method cannot efficiently eliminate the influences of narrow dips on the smoothed profile, resulting in the failure to identify shallow grooves. Moreover, the identified grooves are shallower and narrower than the actual dimensions. This method has been embedded into FAA ProGroove software [12] for groove identification. The third method is the gradient-based method [11]. A dip and spike pair can be obtained when the gradient of each groove is calculated, and the forward and backward traversal technique are used to determine the two endpoint points of each groove. The last method is the geometry contour-based method [13], in which Euler-Bernoulli beam filter is developed to produce a filtered profile, and then the differences between the filtered profiles and the original profiles are calculated. The location of potential dips can be determined based on moving window length and the given threshold, in which one drawback exists: a proper threshold needs to be set to determine the deepest point of the potential dip.

The determination of the two endpoint positions of grooves is critical for the computation of groove dimension. Previous studies indicate there are three methods for the determination of two endpoints of grooves [11,12,13,14]. One is the cluster-based approach, in which the two endpoints of the groove depend on the number of points inside grooves [14]. If the inside-groove points and outside-groove points cannot be determined, inaccurate results would be produced. Another approach is the low-pass filter based method which has been embedded into FAA ProGroove software [12]. The determination of two endpoints of grooves relies on the two intersection points between the raw profile and the filtered profile. Since the low-pass based technique cannot efficiently eliminate the influences of narrow dips, thereby this method is not always effective for determination of two endpoints of grooves. The third method uses the forward and backward traversal techniques to determine the starting and ending points of each groove and produces a decent result in most cases [11,13]. However, this method can fail in some special cases, such as when the gradients vary from negative (positive) to positive (negative) at the location inside grooves.

Although the groove identification algorithm in ProGroove can identify out grooves and joints, the unsatisfactory results are obtained. The authors of [13] presented a heuristic method for groove and joint classification, and a decent result was produced for most cases. Generally, there are three approaches used for groove performance evaluation. The first is to estimate hydroplaning speed based on simulation models. The higher the hydroplaning speed is, the better the groove performance is [9,10,17]. The second approach is to measure runway skid resistance in the field. The higher the friction number is, the better the groove performance is [2,14]. The last is to evaluate groove performance by comparing the measured groove dimension with the standard groove configuration [12,13]. The paper focuses on the last approach to evaluate runway groove performance. 

In summary, current studies on groove identification and measurement still have three limitations: (1) they cannot avoid the presence of fake grooves or noises in the process of attempting to find more true grooves; (2) they may miss some grooves and joints if these thresholds are inappropriately set, and the number of identified grooves and joints has a large variation or fluctuation with the change of parameter settings; (3) they cannot exactly localize the slab joints in the scenarios that have more identified dips within a possible range of next slab joint if the heuristic method is used to determine the slab joint location. To address these issues, the more promising and powerful algorithms should be investigated. To accomplish this goal, the point laser-based profiling equipment developed by the U.S. Federal Aviation Administration (FAA) is employed to collect grooving data on a full-scale runway. Subsequently the slab joint and true grooves are separated out from the identified dips that are produced with the K-mean clustering method and moving window traversal method. Finally, the identification and measurement results from the new method is compared with the algorithm in the ProGroove software [12] and Li’s method [13]. Results indicate that the proposed methodology is more powerful for slab-based runway groove identification and measurement.

## 2. Data Acquisition System

**Figure 1:**
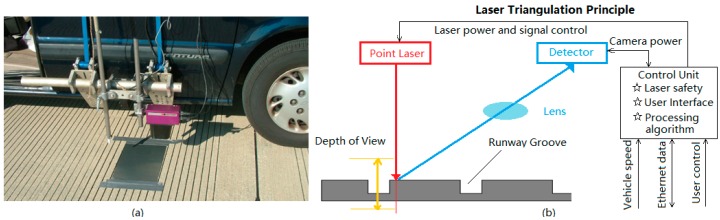
Photos of (**a**) FAA laser-based profiling system (Courtesy of photo in [12]); (**b**) Point laser profiling principle.

FAA point-based laser profiling equipment was developed to conduct the rapid measurement of surface elevation profiles on airport runways, in which an accelerometer and vertical distance sensor are used to measure true changes in concrete or bituminous pavement elevation. Figure 1a shows its exterior appearance. The laser displacement sensor is affixed to a passenger vehicle. The profiler has a laser triangulation-type distance measuring sensor with a nominal spot size of 1 mm (0.04 in), a measurement range of ±200 mm (8 in), a resolution of 12-bits (0.05 mm, 0.002 in), and a sample rate of 32 kHz [18]. One mm resolution profiling data can be obtained with the profiling instrument system as it traverses the roadway surface at speeds of up to 110 km/ h.

Figure 1b demonstrates the wiring of the cameras and lasers to the control unit inside the vehicle. The cameras and lasers are powered by the internal power chassis and triggered by the Distance Measurement Instrument (DMI). The control unit connects to the control computer. The camera and laser working principle can be depicted using Figure 1b as well. A sensor detector is used to capture the laser point from an angle if the laser point is projected onto the grooving surface, and the laser point would be distorted by the surface profile height on the detector. Subsequently, surface profile height can be converted from the distortion using a special algorithm. This capability can capture sufficient accuracy or sample spacing to define the characteristics of transverse grooves on airports. 

With the high-power point laser system, this profiling system can work at highway speeds during daytime and nighttime and maintain data quality and consistency. That means the profiling data can be acquired at any time of the day, regardless of lighting conditions. Even though this system has been designed to accommodate all dynamic and static conditions incurred during normal driving conditions, it cannot collect data on wet runways in rainy days. In addition, the contaminants on runway surface should be cleaned off before data collection, i.e. removal of rubber deposits, removal of sand, dust, mud, etc. It is strongly recommended parking the profiling system either indoors or under certain type of shelter in case of inclement weather since it is not dust-proof and water-tight.

When the profiling system is used for data collection, its parameters or settings should be properly configured, and then the user can open the collection screen and start data collection. The collected data can be plotted in real time in the main widow. The collected data (.dat file) are saved to the designated directory for post-processing. Airport runway surveys are usually conducted at after midnight without any traffic interruption. Typically, each single measurement or pass needs about two minutes depending on airport runway length and the driving speed. The total time for accomplishing the entire runway survey needs about 40 to 60 min depending on the number of measurements or passes.

## 3. Methodology

**Figure 2:**
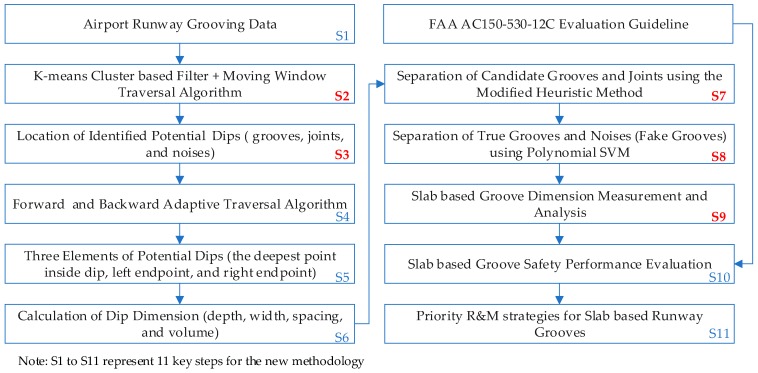
Schematic of the new methodology for concrete slab-based groove identification and performance evaluation.

In previous studies, a four-stage method is proposed for automated groove measurement and evaluation, in which runway grooves can be identified out with Li’s method [13]. However, the identified dips may contain various noises (i.e. cracks), thus the identified groove quantity may have a large variation or fluctuation when compared with the ground truth. We propose a heuristic method in the paper [13] to separate out joints and grooves, nonetheless, the method cannot exactly retrieve the slab joint location for some scenarios due to the limitation of algorithm itself. To address the existing problems, a new methodology is proposed for slab-based runway groove performance evaluation. Compared with the previous study [13], the implementation of the new methodology can be illustrated in Figure 2, among which Steps 2–3 and 7–9 are the major contributions for slab-based groove performance evaluation in this study.

### 3.1. Potential Dip Identification

Identification of runway grooves is dependent on the elevation difference between the raw grooving data and the low-pass filtered data (termed the base curve). A potential dip or candidate groove can be determined if the corresponding elevation difference are greater than a prescribed threshold. Two key problems need to be addressed for the identification of potential dips: (1) the determination of the base curve; (2) the selection of the threshold. 

For grooving data, the generation of base curve is susceptible to impacts of narrow and deep dips, which would lead to the elevation difference at dips are less than the actual dip depths. This phenomenon would bring in the difficulty in identifying shallow or worn grooves if the threshold is given. To solve this issue, K-means clustering based filter is proposed and used to determine the base curve.

#### 3.1.1. K-Means Clustering Based Filter

**Figure 3:**
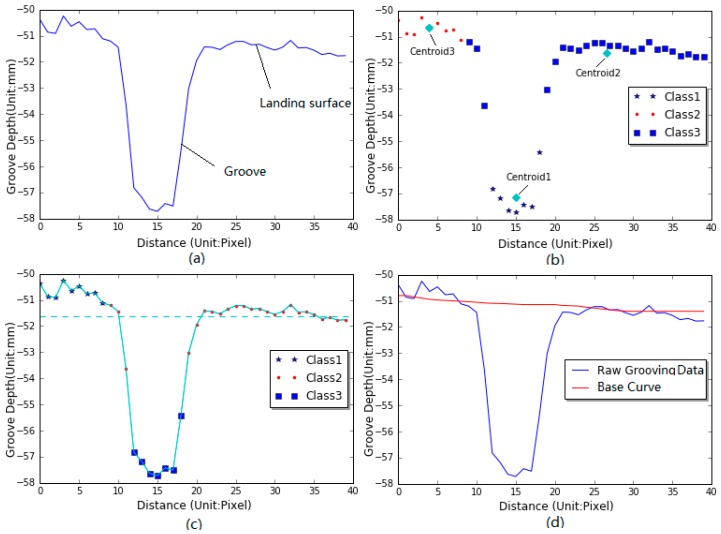
Development and implementation of K-means clustering based filter (**a**) grooving data; (**b**) three clusters; (**c**) data above dashed line used for smoothing; (**d**) the smoothing effect of grooving data.

The basic idea behind the new filter is to partition n observations into k clusters or groups in which each group belongs to the cluster with the nearest mean [19,20]. In this study a set of grooving data $X=(x_{1},x_{2},\cdots ,x_{n})$ (Figure 3a) can be roughly partitioned into three clusters $C=\{C_{1},C_{2},C_{3}\}$, in which it can be assumed that one portion of landing surface points between grooves belong to one class, as circle spots show in Figure 3b. One portion of the inside-dip points can be considered as another class, as asterisks show in Figure 3b, and the remaining points (exclusive of the two classes) can be regarded as the third class, as square spots show in Figure 3b. Therefore, the points consisting of one groove or dip can be roughly divided into three classes or clusters.

Figure 3b shows the three clusters that the grooving data can be classified into. The chosen grooving data is composed of 40 discrete points, and each observation $x_{i}$ (i∈[1,40]) belongs to one of the three clusters $C_{i}$ (i=[1,3]). The determination of each cluster and its centroid can be elaborated as follows:

(1) Assign an initial set of 3-means $\mu_{1}^{\left(1\right)},\mu_{2}^{\left(1\right)},\mu_{3}^{\left(1\right)}$, their values can be randomly chosen from the grooving data X;
(2) Assign each observation to the cluster whose mean has the least squared Euclidean distance, as mathematically described in Equation (1):
  (1) $$C_{i}^{\left(t\right)}=\{x_{i}:||x_{i}-\mu_{i}^{\left(t\right)}|{|}^{2}\leq ||x_{i}-\mu_{j}^{\left(t\right)}|{|}^{2}\;\;\forall j,1\leq j\leq 3\}\;$$
  where each $x_{i}$ is assigned to exactly one cluster $C_{i}^{\left(t\right)}$, $x_{i}$ is the $i^{th}$ observation or grooving data; t is the number of iterations.
(3) Calculate the new means to be the centroids of the observations in the new clusters, as mathematically described in Equation (2):
  (2) $$\mu_{i}^{\left(t+1\right)}=\frac{1}{\left|C_{i}^{\left(t\right)}\right|}\sum_{x_{j}\in C_{i}^{\left(t\right)}}x_{j}\;$$
  where $\left|C_{i}^{\left(t\right)}\right|$ is the number of grooving data in this cluster; $x_{j}$ is the $j^{th}$ grooving data in the cluster $C_{i}^{\left(t\right)}$.

The implementation proceeds by alternating between two steps (2) and (3), and is converged when the within-cluster sum of squares for the chosen set of grooving data are minimized. Finally, the three clusters and their centroids can be determined, as mathematically described in Equation (3):

(3) $$\min\sum_{i=1}^{3}\sum_{j=1}^{j=|C_{i}^{\left(t\right)}|}||x_{j}-\mu_{i}|{|}^{2}\;\;x_{j}\in C_{i}^{\left(t\right)}\;$$

where $\mu_{i}$ is the mean or centroid of grooving points in the cluster $C_{i}^{\left(t\right)}$, $x_{j}$ is the $j^{th}$ grooving data or observation in the cluster $C_{i}^{\left(t\right)}$. The development of the base curve for calculating elevation differences is illustrated in Figure 3. Firstly, a certain range of grooving data are chosen (Figure 3a), and the number of data used for clustering is dependent on dip spaces. Subsequently the chosen grooving data are partitioned into three clusters (Figure 3b) by iterating Equations (1) and (2) until the minimum of the within-cluster sum of squares is reached, and simultaneously the three centroids of clusters can be obtained, Thirdly, the three centroids are ranked in descending or ascending sequence, and the grooving data greater than middle one (the centroid 2 in Figure 3b) is used for the development of base curve by averaging their values, that is, the base curve can be developed by averaging the grooving data above the imaginary line in Figure 3c. Its implementation can be briefly described: (1) find the first point of the grooving data, and determine its centered window with a fixed length (the window length is 40-pixel long); (2) average the grooving data greater than the centroid2, and store the mean value into a new array; (3) keep moving to the next point and perform the same process until all the points of grooving data are processed, and then the new array or base curve having the same size with the raw grooving data can be produced, as shown in Figure 3d. 

**Figure 4:**
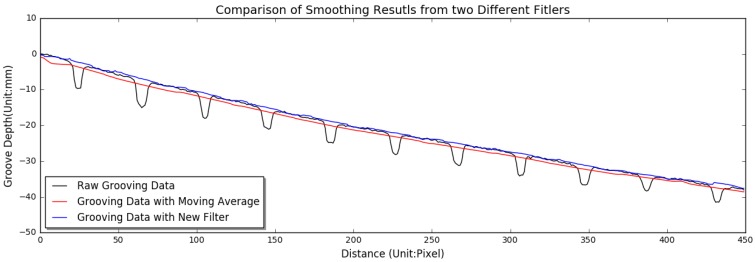
Comparison of smoothing results with moving average filter and the new filter.

Figure 4 demonstrates the effects of moving average filter and the new filter on eliminating influences of narrow dips on base curve. Note that the use of moving average would underestimate the elevation difference between the smoothed data and the raw grooving data, and lead to the difficulty in identifying shallow or worn grooves. However, the impacts of narrow dips on base curve are totally suppressed after K-means clustering filter is applied, in which the actual elevation difference can be calculated.

#### 3.1.2. Moving Window Traversal Algorithm

The new grooving data can be calculated by using raw grooving data to subtract base curve, as mathematically described in Equation (4):

(4) $$h_{d}=f_{r}-f_{b}\;$$

where $h_{d}$ is new grooving data, $f_{r}$ is the raw grooving data, $f_{b}$ is the base curve.

In previous studies, a prescribed threshold is used to identify the potential dips. Usually true grooves may be not successfully identified out if the given threshold is too large, while more fake grooves or noises would be introduced if the given threshold is too small. Accordingly, the selection of threshold is critical for the accurate identification of potential dips. To address this issue, moving window traversal algorithm is proposed to locate the deepest point within each potential dip across the window length (BL). Practically the moving window length is dependent on groove space. 

**Figure 5:**
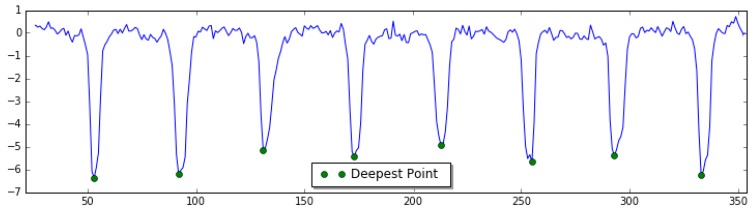
Determination of the deepest point inside dips.

For each point i of the array $h_{d}$, the corresponding window centered at point i can be derived. If the elevation at point i is no greater than the elevations of all points within the window, and simultaneously is less than that of two adjacent points, this point is considered as the deepest point of one potential dip of the new grooving data, as shown in Figure 5. Its mathematical equation is described in Equation (5):

(5) $$\left\{ \begin{array}{l} h_{d}(i)<h_{d}(i-1)\\ h_{d}(i)<h_{d}(i+1)\\ h_{d}(i)\leq h_{d}(j) \end{array}\right.,\;j\in [i-\frac{BL}{2},i+\frac{BL}{2}]\;\&\;j\neq i,i-1,i+1\;$$

### 3.2. Dip Dimension Calculation

The calculation of dip dimension is dependent on the three elements of a dip, namely the deepest point, left endpoint, right endpoint. Therefore, the following task is to find out the two endpoints of a dip once the deepest point is located.

#### 3.2.1. Adaptive Forward and Backward Traversal Technique 

In this study backward traversal technique is used to locate the left endpoint of a dip. Its implementation starts at the deepest point of a dip until the traversed gradient is no smaller than zero, and simultaneously the current pixel value is larger than the corresponding pixel value of the auxiliary curve. The calculation of gradient of a point can be mathematically described in Equation (6):

(6) $$\nabla f=\frac{\partial y}{\partial x}=f(x+1)-f(x)\;$$

**Figure 6:**
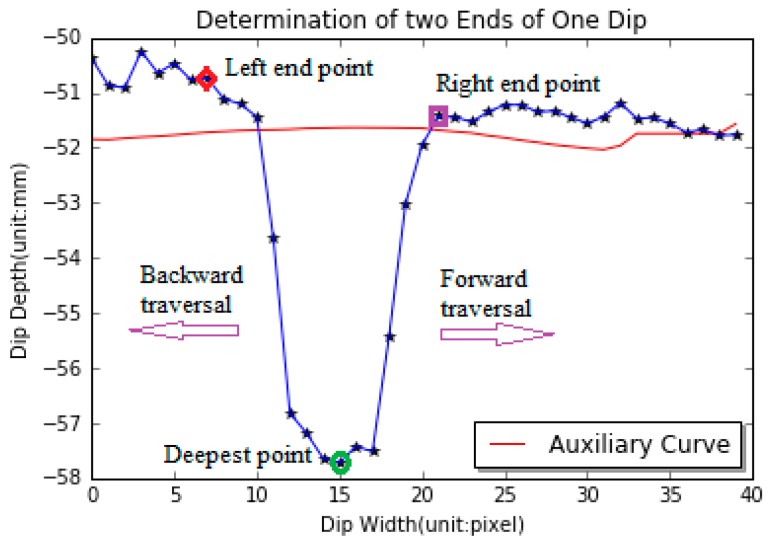
Diagram of determination of two endpoints of a dip.

Similarly, the forward traversal technique is used to find the right end point of a dip, starting at the deepest point of a dip until the traversed gradient is no larger than zero and the current pixel value is larger than the corresponding pixel value of the auxiliary curve. Figure 6 shows the procedure of determination of the two endpoints of a dip. The deepest point is marked as the green circle. Backward traversal operation ends at the red diamonds since the gradient of its previous point is larger than zero, and the point marked by the red diamond can be regarded as the left endpoint of the groove. Forward traversal operation terminates at the point marked by the purple square since the gradient of its next point is less than zero. As a result, the point marked by the purple square is identified as the right endpoint of the groove.

#### 3.2.2. Dimension Calculation Practice

**Figure 7:**
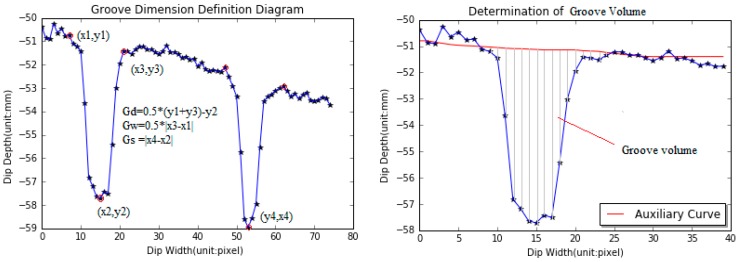
Diagram of calculation of a dip dimension.

Once the deepest point inside dips and the two endpoints of dips are determined, dip dimension can be computed based on the procedure specified in the FAA Advisory Circular No. 150/5320-12C [2]. Groove depth is the elevation difference between the average of the starting and ending point elevations and the deepest point elevation. Groove width is the half of the distance between the starting and ending points. Groove spacing is the distance between the center to the center of two adjacent grooves, as the left plot in Figure 7 shows. 

Groove volume, an important statistical index associated with wet pavement safety, is initially proposed to describe the drainage capacity of measured grooves. Grooving volume equals to the sum of elevation difference between the base curve and raw grooving data for all points inside each groove, as the right plot in Figure 7 shows.

### 3.3. True Groove Determination

The identified dips consist of true grooves, slab joints, and other noises (i.e., cracks). Therefore, true grooves should be distinguished out from the identified dips firstly before they are used for airport runway safety evaluation. This goal can be implemented in two phases: (1) use the modified heuristic method to separate out slab joints from the identified dips, in which the naïve Bayesian classifier is introduced; (2) use the polynomial support vector machine to distinguish true grooves with noises. Finally, the slab-based groove performance evaluation can be performed.

#### 3.3.1. Separation of Slab Joints from Identified Dips

**Figure 8:**
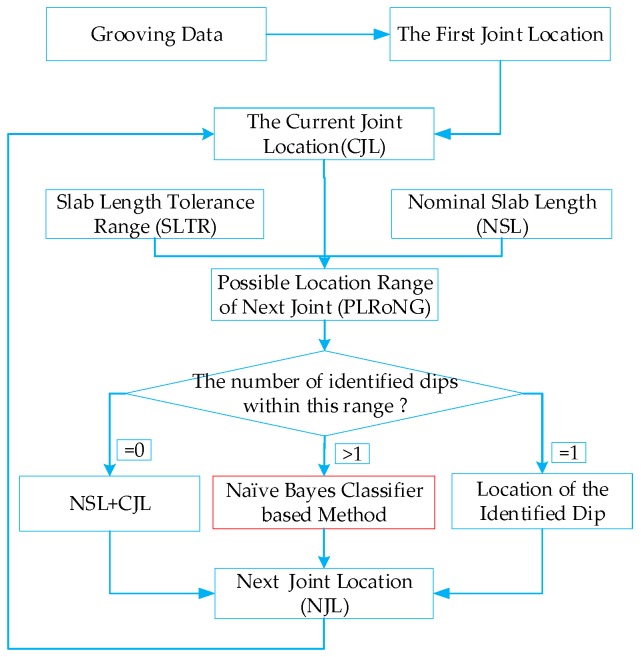
Diagram to separate out slab joints from the identified dips with the modified heuristic method.

To separate out slab joints from the identified dips, the naïve Bayesian classifier is introduced to modify the heuristic method in this study. Its implementation can be described using Figure 8. Firstly, the first joint and its location is automatically determined with the grooving data. Once the first joint location is located, two parameters namely Nominal Slab Length (NSL) and Slab Length Tolerance Range (SLTR) are used to approximately estimate the Possible Location Range of the Next Joint (PLRoNG). If there are no dips falling in the possible range of next joint, the Next Joint Location (NJL) is computed as the Current Joint Location (CJL) plus the NSL. If there is only one dip falling in the possible location range of the next joint, the location of the identified dip is the NJL. If there are multiple dips that fall in possible location range of the next joint, the NJL can be determined with naïve Bayesian classier. The implementation of separation of slab joints from identified dips can be elaborated in Figure 8.

Naïve Bayes is a simple technique for constructing classifiers: models that assign class labels to problem instances, represented as vectors of feature values, where the class labels are drawn from some finite dataset [21,22,23]. The naive Bayes classifiers assume that the value of a particular feature is independent of the value of any other feature. In this study, the identified dip within the possible location range of the next joint may be considered to be a slab joint if it has the reasonable dip depth $x_{d}$, dip width $x_{w}$, left dip spacing $x_{l}$, and right dip spacing $x_{r}$, represented by a feature vector $x=(x_{d},x_{w},x_{l},x_{r})$. Apparently these four variables are independent, and each of them contributes independently to the probability that the identified dip is a slab joint.

The identified dips falling inside the possible range of the next joint can be divided into two classes: the groove or noise $c_{d}$ and the slab joint $c_{j}$, represented by a class vector $c=(c_{d},c_{j})$. Using Bayes’ theorem, the conditional probability can be decomposed as Equation (7):

(7) $$p(c_{k}|x)=\frac{p(c_{k})(x|c_{k})}{p(x)}\;$$

where $c_{k}$ represents the two possible outcomes or classes, namely slab joints ($c_{j}$) and others ($c_{d}$). x represents four features $\left(x_{d},x_{w},x_{l},x_{r}\right)$ of the problem stance to be classified. 

In practice, the identified dip depth, width and volume belongs to a continuous dataset, so a typical assumption is that the continuous values associated with each class are distributed according to a Gaussian distribution. For instance, the training feature $x_{d}$ is segmented by the class $c_{j}$, and then compute the mean ($\mu_{c_{j},d}$) and variance ($\sigma_{c_{j},d}^{2}$) of the feature $x_{d}$ of the class $c_{j}$. Suppose we have collected some observation value $x_{i}$ for feature data $x_{d}$. Then, the probability distribution of $x_{i}$ given a class $c_{j}$, can be mathematically computed in Equation (8):

(8) $$p(x_{i}|c_{j})=\frac{1}{\sqrt{2\pi }\sigma_{c_{j},d}}\exp(-\frac{{\left(x_{i}-\mu_{c_{j},d}\right)}^{2}}{2\sigma_{c_{j},d}^{2}})\;$$

**Figure 9:**
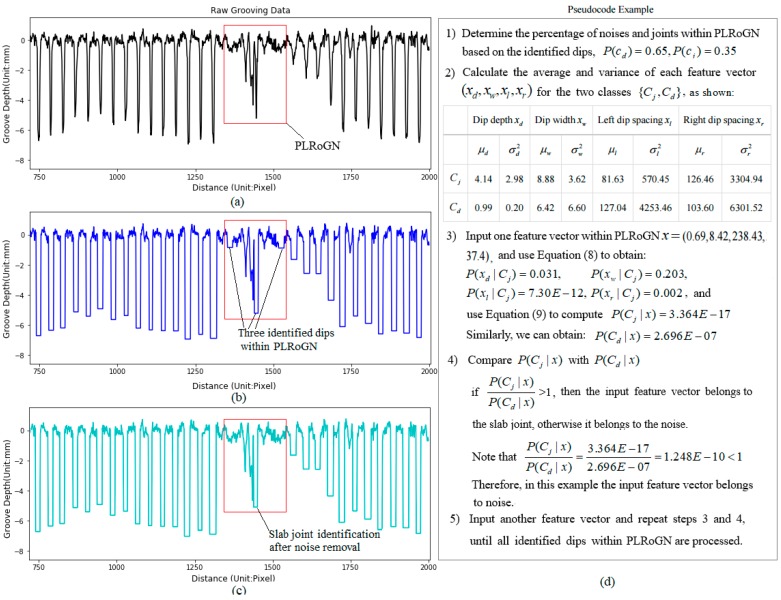
Example for separation of joints and noises: (**a**) raw grooving data; (**b**) identified dips within PLRoGN; (**c**) identified joint after noise removal; (**d**) flowchart for the separation of joints and noises.

In Equation (7) there is interest only in the numerator, because the denominator does not depend on classes $c=(c_{d},c_{j})$ and the values of the features are given, so that the denominator is constant. According to the joint probability model, the numerator in Equation (7) is equivalent to $p(c_{k},x_{d},x_{w},w_{l},w_{r})$, and thus the he corresponding classifier, a Bayes classifier, can be rewritten in Equation (9). The Bayes classifier is the function that assigns a class label for some given features, as illustrated in Figure 9:

(9) $$\hat{y}=\underset{c\in (c_{d},c_{j})}{\arg\max}\;p(c)\prod_{i=1}^{4}p(x_{i}|c)\;$$

In addition, the percentage of the cases where the joints fall or not in the PLRoNG is examined based on the twenty concrete slabs. In this study the NSL equals to the mean (4.46 m) of the measured slab lengths in fields, and SLTR (0.054 m) equals to two times the standard deviation. The findings indicates the chosen twenty slabs all fall in the PLRoNG since the maximal and slab lengths are 4.51 m and 4.41 m, respectively. 

#### 3.3.2. Separation of Noises and Grooves

Once slab joints are distinguished from the identified dips, the following task is to separate out noises and true grooves using Support Vector Machine (SVM) based on the three variables or vectors namely dip depth ($X_{d}$), dip width ($X_{w}$), and dip space ($X_{s}$). The SVM is a state-of-the-art classification method in machine learning and artificial intelligence, especially for solving two-class problems. The SVM model is a representation of the examples as points in space, so that the examples of the separate categories are divided by a clear gap that is as wide as possible [24]. Typically, this clear gap is defined as the hyper plane. Once the hyper plane is located, the new sample is then mapped into that same space and predicted to belong to a classification based on which side of the hyper plane they fall.

**Figure 10:**
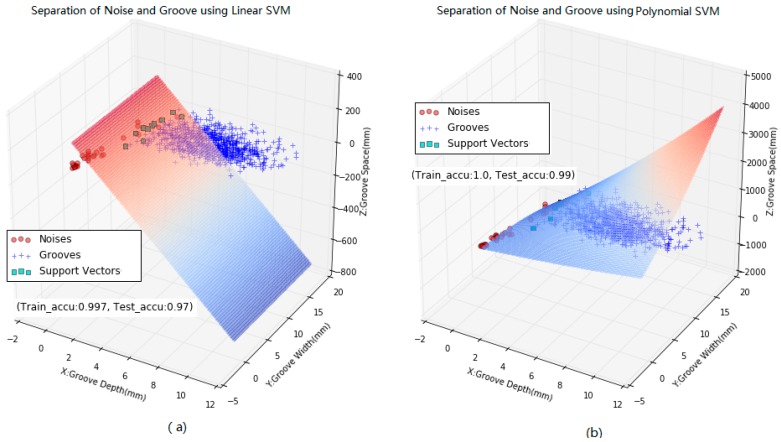
Comparison of separation of noises and true grooves with three feature vectors: (**a**) linear model; (**b**) polynomial model.

SVMs include linear and non-linear classification methods depending on kernel models or functions. Usually there are four types of kernel models, namely linear kernel, polynomial kernel, Gaussian kernel, and sigmoid kernel [25,26,27,28,29,30]. In this study the linear kernel and polynomial kernel are used to validate which method is more powerful in the separation of grooves and noises based on training data and test data. After several trials and errors, the polynomial kernel appears more robust for separating out noises and true grooves, and its implementation effects are given in Figure 10. 

The crux of the PSVM is to map our data from the low dimensional space to a high dimensional space using a non-linear function ϕ. In the high-dimensional space, the hyper plane can be mathematically expressed using Equation (10), in which vector weights W and bias b of hyper plane or discriminant function f(X) should be solved:

(10) $$f(X)=W^{Τ}\phi (X)+b\;$$

To maximize the geometric margin of the hyper plane, the function $\phi (X)=\frac{1}{2}W^{Τ}W$ should be minimized. In practice, data is often not linearly non-separable, indicating the hard margin SVM cannot efficiently separate out noises, grooves and joints. Therefore, slack variables ($\xi_{i}$) are introduced to allow the classifier to misclassify some points by achieving a great margin. In addition, the term $C\sum_{i}\xi_{i}$ is used to penalize misclassification and margin errors, in which the constant C is termed as the penalization factor and its value is no less than zero. Herein, the optimization problem can be written in Equation (11):

(11) $$\underset{W,b}{\min}\;\frac{1}{2}W^{T}W+C\sum_{i}\xi_{i},\;y_{i}(W^{T}\phi (x_{i})+b)\geq 1\;-\xi_{i}\;\xi_{i}\geq 0\;\&\;i=1,\ldots ,n\;$$

It is well known that the Lagrange function is widely used to find optimal solution of a function. In this study it is used for the optimal solutions of $W_{0}$ and $b_{0}$, and its mathematical description can be expressed by Equation (12):

(12) $$\begin{array}{l} L(w,b,\xi ,\alpha ,\mu )=\frac{1}{2}W^{T}W+C\sum_{i=1}^{m}\xi_{i}+\sum_{i=1}^{m}\alpha_{i}(1-y_{i}(W^{T}\phi (x_{i})+b)-\xi_{i})-\sum_{i=1}^{m}\mu_{i}\xi_{i}\\ \;\;\;\;\;\;\;\;\;\;\;\;\;,\alpha_{i}\geq 0,\mu_{i}\geq 0 \end{array}\;$$

where L(w,b,ξ,α,μ) is Lagrange function or expression; $\alpha_{i},\mu_{i}$ are Lagrange multipliers.

To minimize Lagrange function, the calculation of partial derivatives of L(w,b,ξ,α,μ) with respect to vector weights, bias, and slack variables can be mathematically expressed in Equations (13)–(15). Subsequently, the calculated vector weights are given in Equation (13), and a pair of equality constraints are obtained and given in Equations (14) and (15):

(13) $$\frac{\partial L}{\partial w}=0\;\Rightarrow \;w=\sum_{i=1}^{m}\alpha_{i}y_{i}\phi (x_{i})\;$$

(14) $$\frac{\partial L}{\partial b}=0\;\Rightarrow \;\sum_{i=1}^{m}\alpha_{i}y_{i}=0\;$$

(15) $$\frac{\partial L}{\partial \xi_{i}}=0\;\Rightarrow \;\alpha_{i}+\mu_{i}=C\;$$

Equation (13) is used to replace W in Equation (11). According to the Kuhn Tucker theory, the optimal solution for Equation (11) can be deduced and rewritten as Equation (16):

(16) $$\hat{\alpha }=\underset{\alpha }{\arg\max}[\sum_{i=1}^{m}\alpha_{i}-\frac{1}{2}\sum_{i=1}^{m}\sum_{j=1}^{m}\alpha_{i}\alpha_{j}y_{i}y_{j}\phi (x_{i}^{Τ})\phi (x_{j})],\;\sum_{i=1}^{m}\alpha_{i}y_{i}=0,\;0\leq \alpha_{i}\leq C,\;\forall i=1,\cdots ,m\;$$

Assume the optimal Lagrange multiplier is {$\alpha_{0p},\alpha_{1p},\cdots ,\alpha_{0p}$}, the optimal weight vector can be calculated and rewritten as Equation (17), and the optimal bias can be calculated using Equation (18). Once the $W_{0}$ and $b_{0}$ are calculated, hyperplane coefficients can be determined accordingly.

(17) $$\hat{w}=\sum_{i=1}^{m}{\hat{\alpha }}_{i}y_{i}\phi (x_{i}^{})\;$$

(18) $$\hat{b}={\hat{y}}_{j}-{\hat{w}}^{Τ}x_{j}={\hat{y}}_{j}-\sum_{i=1}^{m}{\hat{\alpha }}_{i}y_{i}\phi (x_{i}^{Τ}x_{j})\;$$

where $X^{s}$ is the support vector sample; ASV is defined as all support vectors; $\alpha_{0s}$ is the Lagrange multiplier of the support vector sample $X^{s}$; $Y^{s}$ is the classification label for the support vector sample $X^{s}$.

As a result, the category that contouring box belongs to can be determined based on Equation (19). If the sign of f(X) function is positive, the contouring box belongs to true grooves, otherwise it belongs to noises or fake grooves:

(19) $$f(X)=\mathrm{sgn}(W_{0}^{T}\phi (X)+b_{0})\;$$

### 3.4. Groove Performance Evaluation Guideline

The FAA AC No. 150-5320-12C provides standard groove configuration and the tolerable range in depth, width, and spacing for the entire airport runway or each PCC slab [2]. The standard groove depth is 6.35 mm (±1.78 mm), the groove width is 6.35 mm (0 mm, 1.78 mm), and the spacing is 38.1 mm (−3.55 mm, 0 mm), as illustrated in Table 1. For acceptable groove performance, the depth and width of 90 percent or more of the grooves shall not be less than 4.76 mm; the depth and width of 60 percent or more of the grooves shall not be less than 6.35 mm; the depth and width of 10 percent or less of the grooves shall not be more than 7.94 mm, based on which the slab-based groove performance evaluation can be conducted.

## 4. Experimental Analysis

**Figure 11:**
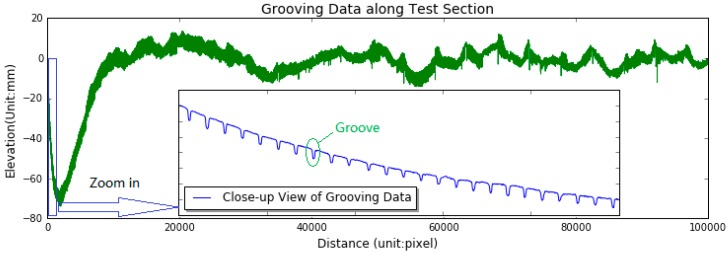
Test grooving data and its close-up view.

One small section of the grooving data, made of 100,000 discrete points, are chosen to illustrate the implementation of automated groove identification, measurement and evaluation. The chosen grooving data provided by FAA has been in service for several years, with a length of approximately 95 m, as shown in Figure 11. The close-up view of a small segment of grooving data is plotted in Figure 11 as well, from which the grooves can be clearly observed. The groove and joint quantity in this section have been manually surveyed by two trained investigators, with the values of 2360 and 21, respectively.

The implementation of groove measurement and evaluation is organized as follows: (1) the potential dips are identified out with the three approaches namely the algorithm in ProGroove software, Li’s method, and the newly proposed method. The comparison results are analyzed based on identification results; (2) the slab joints are distinguished from the identified dips using the modified heuristic method, and then the true grooves are separated out from the candidate grooves using polynomial SVM method. In this study the identified dips include true groove, slab joint, and other noises, whilst the candidate groove contains true groove and other noises; (3) the slab-based groove performance is evaluated, and the corrective measures can be taken on each concrete slab to improve runway skid resistance.

### 4.1. Potential Dip Identification

To position the potential dips along runway grooving data, the K-means clustering based filter and moving window traversal algorithm are initially used to locate the deepest point of each potential dip. Each deepest point that is identified represents one potential dip. Subsequently, adaptive forward and backward traversal algorithms are used to find the left and right endpoints of each identified dip based on the location of the deepest points that are identified. Once the locations of the three elements (the deepest point, the left endpoint, and the right endpoint) of a dip are determined, each potential dip dimension can be computed. The number of identified dips and from the three methods are summarized in Table 2. Note that the ground truth is the sum of true groove quantity and slab joint quantity. Note that the identified dip quantity from the algorithm in ProGroove software is 2512, which is 131 more than the ground truth. The identified dip quantity from the Li’s method is 2239, which is 142 less than the ground truth. With the new method, the identified dip quantity is 2426, which is 45 more than the ground truth.

**Figure 12:**
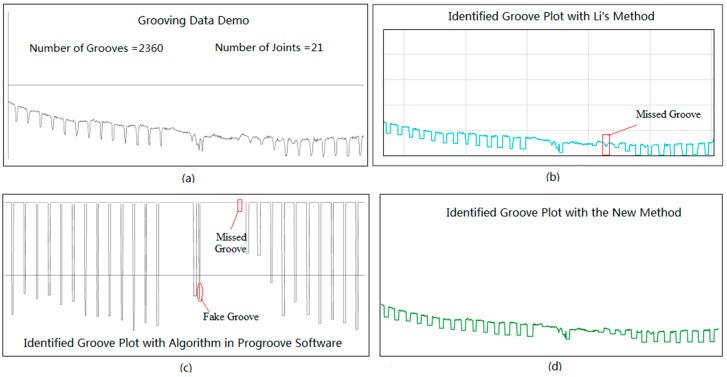
Identification result comparison: (**a**) Original grooving data; (**b**) Missed groove with Li’s Method; (**c**) Missed and fake groove with ProGroove software; (**d**) identified result with the new method.

Figure 12 shows a small part of grooving data and the corresponding identification results with the three methods. The chosen grooving data is illustrated in Figure 12a. Li’s method tends to fail to identify some true grooves, especially for the shallow or worn grooves, as the square marked in Figure 12b indicates. In light of the algorithm in ProGroove software, the increase of the identified dip quantity is the result of the increase of the identified fake grooves or noises, as the circle marked in Figure 12c. On the contrary, the newly proposed method can efficiently suppress the increase of fake grooves and the failed identification of true grooves, and produce a satisfactory identification result, as shown in Figure 12d.

As mentioned above, the identified dips with the three methods contain true grooves, slab joints, and other noises. To analyze the groove dimension distribution on each PCC slab and perform slab-based groove performance evaluation, the slab joint should be determined first.

### 4.2. Slab Based Groove Dimension Measurement

According to the heuristic method used in the paper [13], the slab joint can be easily determined for the scenario that there is one or no dips falling in the possible range of next joint. However, this method would be inefficient for the scenario that has multiple dips falling in the possible range of next joint. To solve this problem, the modified heuristic method is initially used to localize the slab joint, in which naïve Bayes classier is introduced. Table 3 lists the detailed joint identification results with the new method and the Li’s method.

In this experiment, the number of identified joints with the algorithm in ProGroove software is about 319, which is 298 more than the ground truth. Accordingly, the algorithm in ProGroove software is inappropriate for slab joint identification. In light of joint locations, all the slab joints at the test section are identified with the proposed algorithm, and the identified joint locations correspond to the joint locations in field. Li’s method fails to detect two joints. As a result, the newly proposed method is more suitable for the slab-based groove performance evaluation.

Once the slab joint is determined, the subsequent work is to separate out noises or fake grooves from the candidate grooves on each individual slab, and the polynomial SVM is used to achieve this goal. To quantitatively describe identification results of candidate grooves, three evaluation metrics namely precision, recall, and F-score are presented in this study [31]. For each groove, it can be regarded as “True Positive” (TP) if the automatic identification result exactly matches with the manual survey result (ground truth); otherwise, it would be regarded as the “False Negative” (FN). For non-groove, it can be considered as “True Negative” (TN) if the identified fake groove (joint or noise) still is fake groove; otherwise, it would be considered as the “False Positive” (FP). In this study TP and TN are regarded as the acceptable identification results, while FP and FN are considered as the unacceptable identification results. 

Once the TP, TN, FP, and FN are determined, three evaluation metrics can be calculated, as described in Equations (20)–(22). Generally, the larger the evaluation metrics is, the better the performance of the test algorithm is. An ideal or robust algorithm would have values of all evaluation metric approximating to one:

(20) Precision=TP/(TP+FP) 

(21) Recall=TP/(TP+FN) 

(22) $$F=\frac{2\times Precision\times Recall}{Precision+Recall}\;$$

The goal of the test algorithm is to be the precision and recall close to 1.0. Table 4 shows groove identification results with the algorithm in ProGroove software, which indicates that improvements can be achieved due to the lower recalls with a value of 0.88. The groove identification results with the precision (0.99) and recall (0.94) are obtained for Li’s method. Table 4 also lists the identified groove quantity from new methodology, with the precision of 1 and the recall of 0.96. Results indicate the new method can produce a better precision in groove identification by eliminating the fake grooves.

**Figure 13:**
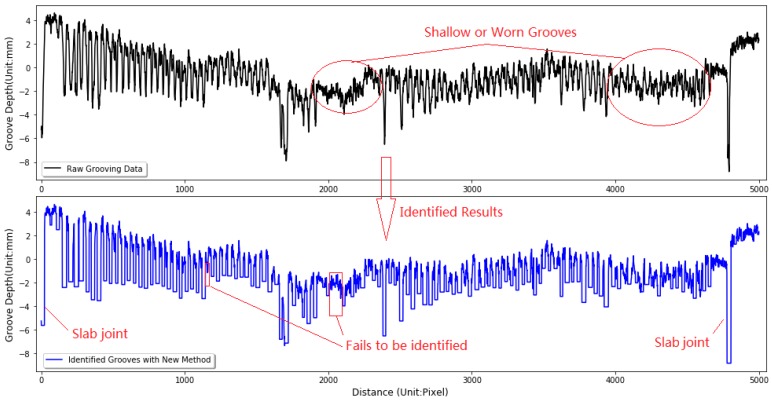
Plots of the severely worn grooves on slab 17 and their identification results.

However, the three methods produce the lower recalls when compared with their respective precisions. Figure 13 shows the grooving data and the identification result on Slab 17, from which it can be observed that some severely deteriorated grooves appear, as circled in Figure 13. For these severely worn grooves, it would produce the lower recalls when the automatic identification methods are used. Actually, it is also difficult to find the severely worn grooves even with manual methods. As a result, the comparatively lower recalls are produced due to the presence of the severely worn grooves.

### 4.3. Slab based Groove Performance Evaluation

To conduct groove performance evaluation on each individual slab, it needs to report the statistical groove information for each individual slab based on classification results of grooves and joints. The measured groove dimension (depth, width, spacing and volume) and the three-evaluation index (percentages of groove depths >4.76 mm, percentages of groove depths >6.35 mm, percentages of groove depths >7.94 mm) with the proposed approach are summarized in Table 5. 

**Figure 14:**
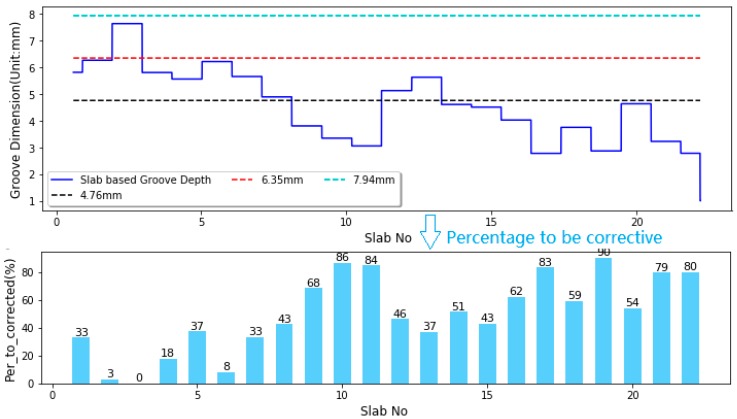
Plots of the groove depths along runway slabs and the corresponding percentage of grooves that need to be maintained.

According to the FAA AC No. 150-5320-12C guideline, there are no slabs that conform to the guideline requirement. For Slab3, although the percentages of grooved depths larger than 4.76 mm is over 90% and that larger than 6.35 mm is over 60%, the percentage of groove depths larger than 7.94 mm is more than 10%, which do not meet guideline requirement. For other slabs, the percentages of groove depths larger than 6.35 mm are less than 60%, which do not meet guideline requirements. To make grooves on each slab meet the FAA guideline requirement, part of grooves on each slab need to be corrected, as illustrated in Figure 14. The percentage or quantity of grooves that need to be corrected on each concrete slab can be computed in Equation (23):

(23) $$P_{c}=\max((90\%-p_{4.76}),(60\%-p_{6.35}),0)\;$$

where $P_{c}$ represents the percentage of slab-based grooves that need to be maintained; $p_{4.76}$ represents the percentage of slab-based grooves with the depth ≥4.76 mm; $p_{6.35}$ represents the percentage of slab-based grooves with the depth ≥6.35 mm.

The corrective activity aims to increase groove depths at test runways except for the Slab No 3, and to ensure groove depths are above the low limit of acceptable range. If the slabs after maintenance have the standard groove configuration, all evaluation indices comply with the FAA standard, which means the groove performance on this section is able to conform to the FAA guidelines once the 21 slabs (exclusive of Slab No 3) are maintained with corrective measures. Accordingly slab based evaluation method is able to provide airport authority with the slab number and also the percentage of slabs that need corrective actions. 

**Figure 15:**
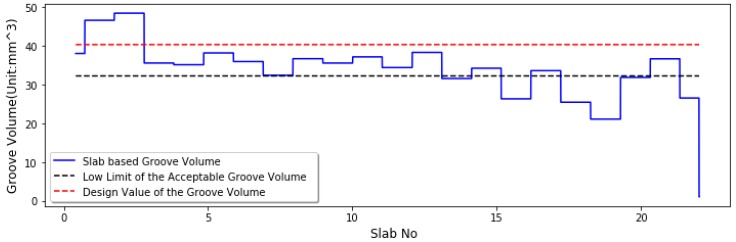
Plot of the groove volume distribution along runway test.

However, groove depth cannot efficiently reflect the drainage ability of the test grooves. For instance, if two grooves have the same depths, they should have the same performance in accordance with the FAA groove evaluation guideline, but actually the two grooves could have the different drainage capacity due to the different groove volumes. As a result, the groove volume-based evaluation method is initially proposed for groove performance evaluation in this paper. Figure 15 shows the groove volume distribution along runway test. The low limit of the acceptable groove volume is 30.23 mm^3^, which is computed as the product of the lower limit of groove depth by the design value of groove width. The design value of groove volume is 40.32 mm^3^, which is computed as the product of the standard groove width by the standard groove depth. 

Note that the measured groove volume is less than the acceptable volume value (30.23 mm^3^), for Slabs No. 14, 16, 18–20, and 22, indicating the test runway grooves are not efficient for rapid discharge of the rainy water on the pavement surface during wet weather. Before any corrective measures are taken, the groove depths should be investigated. If the groove depth distribution does not meet the FAA guideline requirement, the corrective measures are taken to increase groove depth. If the groove depth distribution satisfies the guideline requirement, the cleaning work should be taken to sweep away the surface debris or loosened aggregate inside grooves and to ensure runway surface grooves have the good drainage ability during wet weather.

## 5. Conclusions and Recommendations

In this study a new methodology for automatic groove identification, measurement, and evaluation is proposed. First, experimental data are collected using the FAA point laser-based profiling equipment. The K-means cluster filter and moving window traversal algorithm are developed to identify the deepest point of each potential dip, and then the backward and forward transversal methods are used to position the left and right endpoints of the potential dip. Once the three elements of a dip are determined, the dip dimension is computed. Subsequently, the modified heuristic method is initially used to separate out slab joints from the identified dips, and the polynomial SVM is proposed to distinguish out the true grooves from the candidate grooves. The groove identification results with the new method are compared with that of the algorithm in ProGroove and the Li’s method, findings show the new method is more powerful in groove and slab identification, with the precision of 1.0, the recall of 0.96, and the F-measure of 0.98. Eventually, slab-based groove dimension performance can be evaluated in accordance with the FAA Advisory Circular AC 150-5320-12C. In addition, the groove volume-based evaluation approach is also proposed in this study, which would be beneficial in complimenting the existing runway groove performance methods. Runway pavement engineers can use the evaluation results as basis to take corrective measures on slabs with poor groove performance. 

Even though the new method produces the better precision and recall in groove identification than other existing methods, computation cost is expensive in k-means clustering, and usually it would take a longer time than other methods to process the same amount of data. Moreover, the point laser profiling instruments only collect one line-of-sight profile, which cannot reflect the entire runway groove information due to the limited quantity of profiles. In the future the line-scan imaging data can be used in lieu of point laser-based profiling system to conduct full runway measurement. To increase the computational efficiency, a new filter can be developed to replace the k-mean clustering-based filter in this method. The method presented in the paper can also be extended to the longitudinal or transverse groove performance evaluation for highway applications.

## Figures and Tables

**Table 1:** Recommended Groove Configuration and the Tolerable Range [2]**.**

Groove Type	Standard Configuration (Unit: mm)		Tolerance (Unit: mm)		Acceptable Range	
			Low Limit	Unit: mm	(Unit: mm)	
**Rectangular**	Depth	6.35	−1.78	1.78	4.76	7.94
	Width	6.35	0	1.78	6.35	7.94
	Space	38.1	−3.55	0	34.9	38.1

**Table 2:** Identified Dip Quantity Comparison with three Different Methods.

Identification Quantity	Algorithm in ProGroove	Li’s Method	New Method	Ground Truth
**Potential Dip**	2512	2239	2426	2381

**Table 3:** Joint Identification and Location with Two Different Methods.

ID.	New Method		Li’s Method	
	Joint Location (m)	Dip Type	Location Distance (m)	Description
1	1.36	Joint	1.35	Joint
2	5.77	Joint	5.78	Joint
3	10.24	Joint	10.25	Joint
4	14.72	Joint	14.69	Joint
5	19.19	Joint	19.18	Joint
6	23.65	Joint	23.65	Joint
7	28.10	Joint	28.10	Joint
8	32.57	Joint	32.57	Joint
9	37.04	Joint	37.06	Joint
10	41.54	Joint	41.53	Joint
11	45.95	Joint	45.95	Joint
12	50.46	Joint	50.46	Joint
13	54.90	Joint	N/D (Not Detected)	
14	59.36	Joint	59.36	Joint
15	63.80	Joint	63.93	Joint
16	68.26	Joint	68.32	Joint
17	72.74	Joint	72.73	Joint
18	77.22	Joint	77.21	Joint
19	81.72	Joint	81.70	Joint
20	86.14	Joint	86.07	Joint
21	90.60	Joint	N/D (Not Detected)	

**Table 4:** Comparison of Groove Quantity Measurement Results.

Statistical Descriptor	Algorithm in ProGroove Software	Li’s Method	New Method	Ground Truth
**Identified Dip Quantity**	2512	2239	2426	2381
**Groove Quantity**	2193	2220	2278	2360
**Precision**	0.98	0.99	1	1
**Recall**	0.88	0.94	0.96	1
**F-measure**	0.92	0.97	0.98	1

**Table 5:** Groove Dimension Measurement and Statistic Results with the New Method.

Slab No	Groove Dimension				Evaluation Index		
	Ave_Depth	Ave_Width	Ave_Space	Ave_Volume	>4.76 mm (%)	>6.35 mm (%)	>7.94 mm (%)
1	5.82	7.25	38.08	38.03	87.88	27.27	0.00
2	6.27	7.85	37.86	46.60	90.35	57.02	5.26
3	7.64	7.07	38.13	48.44	98.28	87.93	40.52
4	5.81	7.26	38.21	35.56	72.17	46.96	7.83
5	5.56	7.34	39.14	35.13	53.04	22.61	20.00
6	6.22	7.19	38.09	38.17	90.52	51.72	7.76
7	5.66	7.59	38.30	35.95	65.49	26.55	17.70
8	4.89	7.74	38.10	32.39	47.41	19.83	3.45
9	3.81	9.08	39.18	36.68	21.62	1.80	0.00
10	3.35	9.39	39.02	35.55	3.54	0.88	0.00
11	3.06	10.38	39.89	37.15	5.50	0.92	0.00
12	5.13	7.44	38.18	34.39	43.97	30.17	18.97
13	5.63	7.37	37.93	38.30	87.93	23.28	0.00
14	4.61	6.99	38.80	31.56	38.74	13.51	5.41
15	4.51	8.23	50.06	34.22	61.36	17.05	0.00
16	4.03	7.56	40.22	26.31	27.88	14.42	7.69
17	2.78	10.00	50.77	33.60	6.90	0.00	0.00
18	3.75	7.47	38.24	25.44	30.91	8.18	0.00
19	2.87	7.53	43.17	21.08	0.00	0.00	0.00
20	4.64	7.10	39.30	31.85	36.04	18.02	1.80
21	3.23	10.13	51.64	36.65	10.71	3.57	1.19
22	2.78	8.95	47.15	26.53	10.17	0.00	0.00
